# SARS-CoV-2 proteins and anti-COVID-19 drugs induce lytic reactivation of an oncogenic virus

**DOI:** 10.1038/s42003-021-02220-z

**Published:** 2021-06-03

**Authors:** Jungang Chen, Lu Dai, Lindsey Barrett, Jennifer James, Karlie Plaisance-Bonstaff, Steven R. Post, Zhiqiang Qin

**Affiliations:** 1 Department of Pathology, Winthrop P. Rockefeller Cancer Institute, University of Arkansas for Medical Sciences, Little Rock, AR USA; 2 Department of Medicine, Louisiana State University Health Sciences Center, Louisiana Cancer Research Center, New Orleans, LA USA

**Keywords:** Virology, Infection

## Abstract

An outbreak of the novel coronavirus SARS-CoV-2, the causative agent of Coronavirus Disease-2019 (COVID-19), a respiratory disease, has infected almost one hundred million people since the end of 2019, killed over two million, and caused worldwide social and economic disruption. Because the mechanisms of SARS-CoV-2 infection of host cells and its pathogenesis remain largely unclear, there are currently no antiviral drugs with proven efficacy. Besides severe respiratory and systematic symptoms, several comorbidities increase risk of fatal disease outcome. Therefore, it is required to investigate the impacts of COVID-19 on pre-existing diseases of patients, such as cancer and other infectious diseases. In the current study, we report that SARS-CoV-2 encoded proteins and some currently used anti-COVID-19 drugs are able to induce lytic reactivation of Kaposi’s sarcoma-associated herpesvirus (KSHV), one of major human oncogenic viruses, through manipulation of intracellular signaling pathways. Our data indicate that those KSHV + patients especially in endemic areas exposure to COVID-19 or undergoing the treatment may have increased risks to develop virus-associated cancers, even after they have fully recovered from COVID-19.

## Abstract (web summary)

Chen et al. find that SARS-CoV-2 encoded proteins and some anti-COVID-19 drugs can induce lytic reactivation of Kaposi’s sarcoma-associated herpesvirus (KSHV), one of the major human oncogenic viruses. This study suggests that KSHV-positive patients exposed to COVID-19 or undergoing its treatment may have increased risks to develop virus-associated cancers, even after they have fully recovered from COVID-19.

## Introduction

COVID-19 has become a devastating pandemic since its origin in the city of Wuhan, Hubei province of China in December 2019^1–4^. Based on the updated information (until January 25, 2021) from Johns Hopkins Coronavirus Resource Center (https://coronavirus.jhu.edu/), there are now almost one hundred million global confirmed cases and over 2 million deaths. In the United States, the confirmed cases and deaths have reached 25 million and 420,000, respectively. In addition, the COVID-19 pandemic has caused a huge economic loss due to the necessary shut down and quarantine procedures. Further, many countries have seen secondary spikes in case numbers after reopening their economies. Septic shock and multiple organ failure represent the most common immediate causes of death in patients with severe COVID-19. These deaths are mostly due to suppurative pulmonary infection, onset of a cytokine storm, and the direct attack on multiple organs^5^. Several comorbidities, such as hypertension, cardiovascular disease, endocrine disorder, diabetes, and obesity may increase the likelihood of death from COVID-19 infection^6^. Therefore, it is very meaningful to investigate the impact of COVID-19, including its relationship to pre-existing diseases, including cancer and other infectious diseases.

KSHV is the etiologic agent of several human cancers including Kaposi’s sarcoma (KS), primary effusion lymphoma (PEL), and multicentric Castleman’s disease (MCD)^7,8^, which are mostly seen in immunosuppressed patients. KS is an endothelial-originated multicentric malignant neoplasm, while PEL and MCD represent two types of B-cell lineage disorders^9^. This oncogenic virus belongs to the human γ-herpesvirus subfamily and has two alternating life-cycle programs following primary infection in host cells, the latent and lytic phases^10^. During latency, viral genomes persist as circular episomes with no progeny virion produced and only a limited number of latency-associated genes are expressed. After entering the lytic phase, almost all viral genes are expressed, followed by the replication and release of mature virions. Recent findings indicate that both viral latent and lytic proteins play a pivotal role in the initiation and progression of virus-associated cancers^11^. Here we seek to understand if COVID-19 infection and its related treatments affect KSHV replication and increase the risk of developing virus-associated cancers, and if so how these mechanisms work.

## Results and discussion

**Fig. 1:**
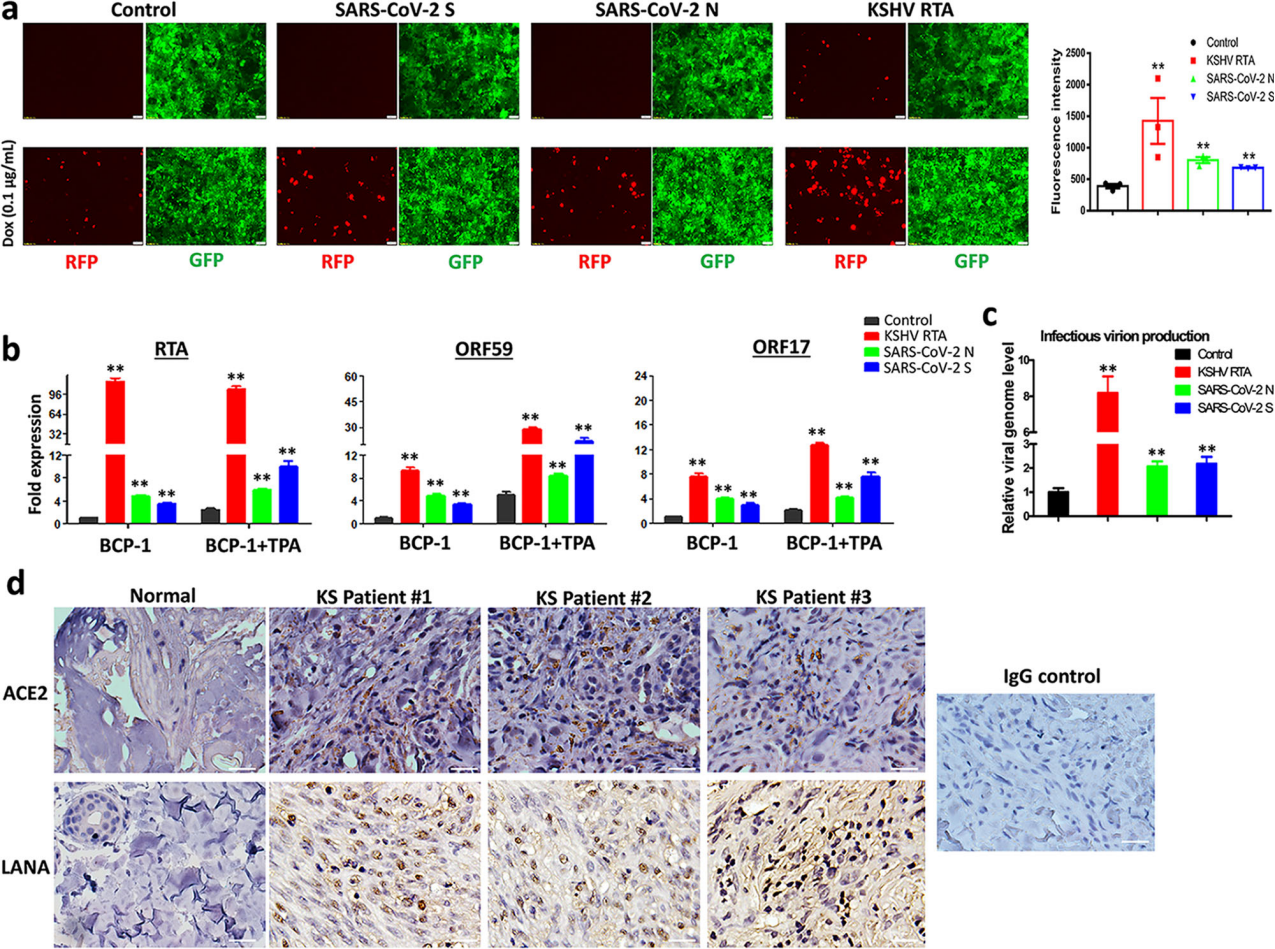
Ectopic expression of SARS-CoV-2 proteins induces KSHV lytic gene expression and ACE2 expression is increased in AIDS-KS tissues. **a** The iSLK.219 cells were transfected with vector control or vectors encoding SARS-CoV-2 spike protein (S), nucleocapsid protein (N) and KSHV RTA (as a positive control) with or without low dose of doxycycline (Dox, 0.1 µg/mL) induction for 72 h. The expression of RFP (representing viral lytic reactivation) and GFP (representing infected cells) were detected using fluorescence microscopy. Error bars represent S.D. for 4 image fields per well. **b**, **c** BCP-1 cells were transfected as above with or without low dose of 12-*O*-tetradecanoyl-phorbol-13-acetate (TPA, 1.0 ng/mL) induction for 72 h, then the transcripts of representative lytic genes were quantified by using qRT-PCR. The supernatants from transfected cells were collected to infect naive HEK293T cells, then viral genome levels were quantified by using qPCR with *Lana*-specific primers. Error bars represent S.D. for 3 independent experiments, **p < 0.01 (vs the vector control). **d** Expression of ACE2 and LANA (as well as representative IgG control) in formalin-fixed paraffin-embedded KS tissues from 3 HIV+ patients and normal skin tissues were determined by immunohistochemical staining as described in the “Methods”. Bars: 50 μm.

The human iSLK.219 cell line carries a recombinant rKSHV.219 virus encoding a constitutive GFP reporter and an RTA-inducible RFP reporter in the viral genome, thereby facilitating the monitoring of viral maintenance and lytic reactivation^12^. To test the potential impact of SARS-CoV-2 on KSHV replication, iSLK.219 cells were transfected with a vector control or vectors encoding two of SARS-CoV-2 major structural proteins, spike protein (S) and nucleocapsid protein (N), and KSHV-RTA (a key viral protein controlling the latency to lytic switch, used as a positive control), and treated during a 72 h induction with or without low dose of doxycycline (Dox at 0.1 µg/mL). The ectopic expression of SARS-CoV-2 S or N, and KSHV RTA proteins were confirmed by immunoblots (Supplementary Fig. 1a-b). Without Dox induction, transfection of KSHV-RTA induced only a small percentage of cells to undergo lytic reactivation. In contrast, induction with a low dose of Dox induction significantly increased lytic reactivation in cells transfected with either SARS-CoV-2 S or N vectors when compared to vector control, although reactivation was a little less than that of KSHV-RTA transfected (Fig. 1a). To confirm these results, we transfected the same vectors into BCP-1, a KSHV + PEL cell line, with or without TPA induction, and confirmed the ectopic expression of SARS-CoV-2 proteins in PEL cells by immunoblots (Supplementary Fig. 1c). Our qRT-PCR data showed that transfection of either SARS-CoV-2 S or N vectors significantly increased representative lytic gene expression (Immediate-early gene, RTA; Early gene, ORF59; and Late gene, ORF17) when compared to vector control, regardless of TPA induction (Fig. 1b). Our results further indicated that transfection with either SARS-CoV-2 S or N vectors significantly increased production of mature virions, which were detected using an infectivity assay in which the supernatants from transfected cells were used to infect HEK293T cells then viral genome levels were quantified by qPCR (Fig. 1c). Considered together, these data indicate that SARS-CoV-2 has the potential to induce KSHV lytic reactivation from latently infected cells. SARS-CoV-2 infects human cells through interaction with the Angiotensin-converting enzyme 2 (ACE2) receptor^13^. Interestingly, our immunohistochemistry staining data (Fig. 1d) indicated that the expression of ACE2 was upregulated in AIDS-KS tissues when compared to that in normal skin tissues, and that many “spindle tumor cells” (LANA+) strongly expressed ACE2.

**Fig. 2:**
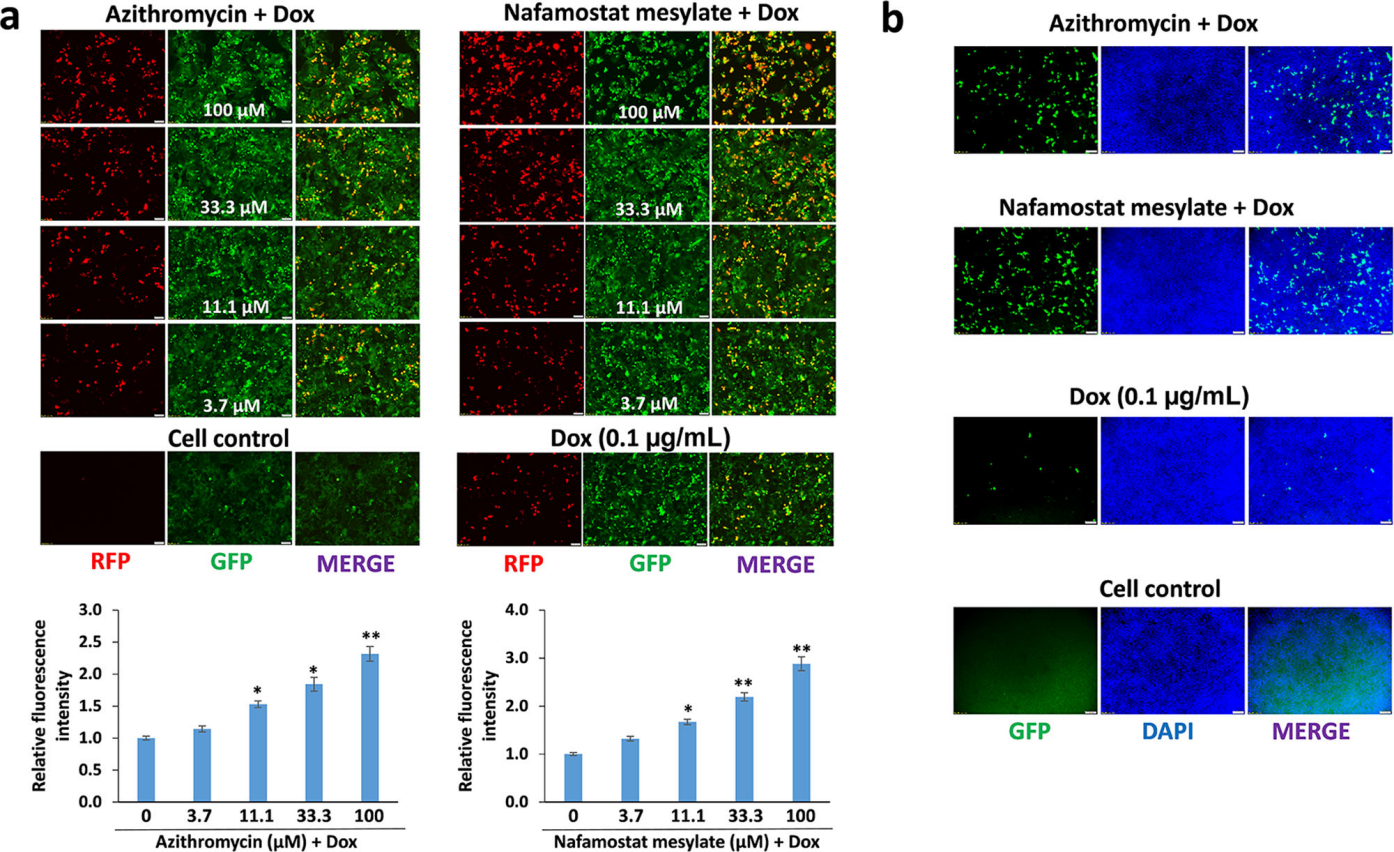
Some drugs currently used for anti-COVID-19 treatment are able to induce KSHV lytic reactivation. **a** The iSLK.219 cells were treated with a dose range of anti-COVID-19 drugs, Azithromycin and Nafamostat mesylate, with doxycycline (Dox, 0.1 µg/mL) induction for 72 h. The expression of RFP and GFP was detected using fluorescence microscopy. Error bars represent S.D. for 4 image fields per well, *p < 0.05; **p < 0.01 (vs the vehicle control). **b** The supernatants from iSLK.219 cells treated by Azithromycin or Nafamostat mesylate for 72 h (11.1 µM, respectively) in combination with Dox were collected to infect naive HEK293T cells, then GFP expression was detected using fluorescence microscopy.

**Fig. 3:**
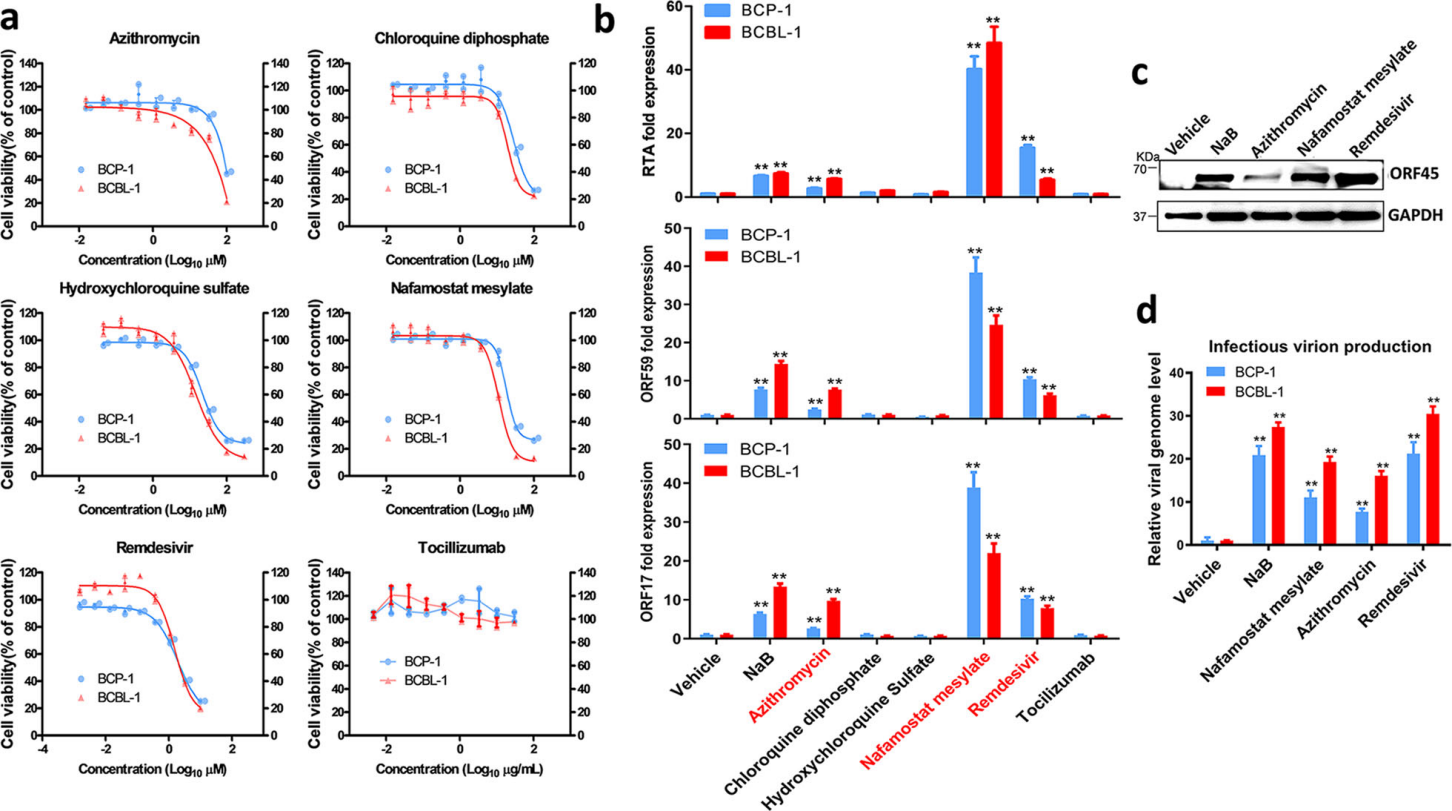
The impacts of anti-COVID-19 drugs on the growth and viral gene expression of KSHV+ tumor cells. **a** BCBL-1 and BCP-1 cells were treated with a dose range of 6 anti-COVID-19 drugs, for 72 h, then the cell proliferation status was examined using the WST-1 cell proliferation assays (Roche). **b**, **c** Cells were treated with Azithromycin (10 µM), Chloroquine diphosphate (10 µM), Hydroxychloroquine sulfate (10 µM), Nafamostat mesylate (10 µM), Remdesivir (3 µM), Tocilizumab (20 µg/mL), respectively, for 72 h, then the transcripts of representative lytic genes were quantified by using qRT-PCR. Protein expression was measured by immunoblots. **d** The supernatants from drug-treated cells above were collected to infect naive HEK293T cells, then viral genome levels were quantified by using qPCR with Lana-specific primers. The sodium butyrate (NaB, 0.3 mM) was used as a positive control. Error bars represent S.D. for 3 independent experiments, **p < 0.01 (vs the vehicle control).

We next examined whether several drugs currently used for treatment of COVID-19 patients may affect KSHV lytic reactivation. A total of 6 drugs were used in our study, including Azithromycin, Chloroquine diphosphate, Hydroxychloroquine sulfate, Nafamostat mesylate, Remdesivir, and Tocilizumab^14–17^. We treated iSLK.219 cells with and without Dox induction to screen these drugs and found that two of these drugs, Azithromycin (an antibiotic) and Nafamostat mesylate (a synthetic serine protease inhibitor), induced KSHV lytic reactivation in a dose-dependent manner with low dose Dox induction (Fig. 2a and Supplementary Fig. 2). Importantly, both Azithromycin and Nafamostat mesylate treatment promoted production of mature virions. As shown in Fig. 2b, the GFP signal in HEK293T cells demonstrates successful production of infectious virions. These drugs were also tested on two KSHV + PEL cell lines, BCBL-1 and BCP-1, and each of the drugs except Tocilizumab inhibited cell growth at high concentrations (Fig. 3a). Using non-toxic concentrations, we found that both Azithromycin and Nafamostat mesylate significantly increased viral lytic (but not latent) gene expression from both PEL cell lines (Fig. 3b–c and Supplementary Fig. 3). Surprisingly, Remdesivir (an adenosine analog) displayed similar effects in the PEL cell lines, which were not seen in iSLK.219 cells. Furthermore, these three drugs significantly increased production of infectious virions from the PEL cell lines (Fig. 3d).

**Fig. 4:**
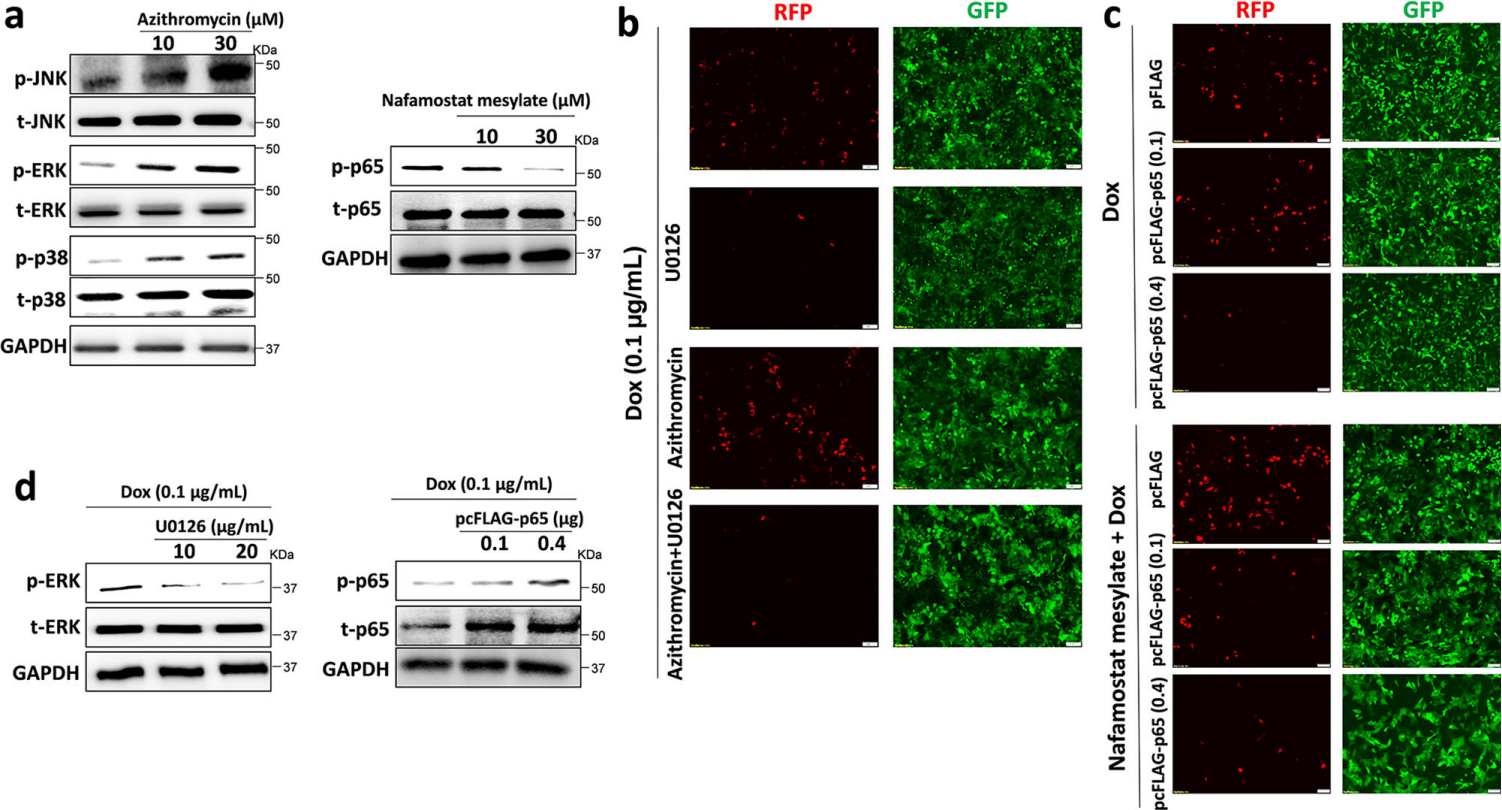
Cellular mechanisms of Azithromycin and Nafamostat mesylate induced KSHV lytic reactivation. **a** BCP-1 cells were treated with indicated concentrations of Azithromycin or Nafamostat mesylate for 72 h, then protein expression was measured by immunoblots. **b** The iSLK.219 cells were pre-treated with U0126 (10 μg/mL), a MAPK kinase inhibitor, for 12 h, then addition of Azithromycin together with doxycycline (Dox, 0.1 µg/mL) induction for 72 h. **c** The iSLK.219 cells were transiently transfected with a construct expressing NF-κB p65 (pcFLAG-p65 at 0.1 or 0.4 μg, respectively) or control vector, then addition of Nafamostat mesylate together with Dox induction for 72 h. The expression of RFP and GFP was detected using fluorescence microscopy. **d** The effects of U0126 and pcFLAG-p65 transfection on signaling activities were confirmed by immunoblots. Representative blots from one of two independent experiments were shown.

KSHV lytic reactivation is reportedly regulated by activation of intracellular signaling pathways including MAPK and NF-κB^8^. We found that treatment with Azithromycin upregulated MAPK signaling activities (increased phosphorylation of JNK, ERK, and p38 kinases), while Nafamostat mesylate treatment downregulated NF-κB activity (reduced phosphorylation of p65) from KSHV + PEL cells (Fig. 4a). To determine if these signaling pathways mediate viral lytic reactivation induced by anti-COVID-19 drugs, we pre-treated iSLK.219 cells with U0126, a specific MEK/ERK inhibitor. We found that Azithromycin-induced viral lytic reactivation was dramatically blocked by U0126 (Fig. 4b). Next, iSLK.219 cells were transfected with a construct expressing NF-κB p65 (pcFLAG-p65 at 0.1 or 0.4 μg, respectively), which effectively blocked Nafamostat mesylate-induced viral lytic reactivation in a dose-dependent manner when compared to vector control (Fig. 4c). The effects of U0126 and pcFLAG-p65 transfection on activation of signaling pathways were confirmed by immunoblots (Fig. 4d). Furthermore, treatment with TNF-α, a strong NF-κB activator, also reduced viral lytic reactivation induced by Nafamostat mesylate (Supplementary Fig. 4). Together, these data demonstrate that anti-COVID-19 drugs induce KSHV lytic reactivation from latently infected cells by regulating the activation of intracellular signaling pathways.

In the current study, we report for the first time that SARS-CoV-2 encoded proteins and some anti-COVID-19 drugs currently used (e.g., Azithromycin and Nafamostat mesylate) induce lytic reactivation of KSHV, one of the major human oncogenic viruses. These events may facilitate KSHV dissemination and initiate viral oncogenesis in those KSHV+ patients exposed to and treated for COVID-19, especially in the case of immunosuppressed patients. Therefore, these patients should be monitored for KSHV viral load and risk of developing virus-associated malignancies, even after they have fully recovered from COVID-19. There are a few limitations and unanswered questions in our study. First, we did not infect cells with the live virus of SARS-CoV-2 because we do not have access to the virus. However, because the S and N proteins represent major and abundant structural proteins of SARS-CoV-2, it is expected that the live virus would display similar effects on KSHV induction. Second, because the KSHV test is not a routine examination in COVID-19 patients, clinical data are not yet available to support our findings. Further, the seroprevalence of KSHV infection in the general population of the United States is less than 10%, but in most of sub-Saharan Africa, the overall seroprevalence is more than 50%^18^. Since mother-to-child transmission of KSHV through saliva is the most common route of transmission, there is a high prevalence of early childhood KSHV infection in some areas^19–21^. Moreover, KS has now become one of the most common overall childhood cancers throughout the central, eastern, and southern regions of Africa^22^. As we know, children are also susceptible to SARS-CoV-2 infection, although with milder symptoms and lower mortality rates^23^. In addition, the seroprevalence of KSHV infection is greatly increased in other sub-populations such as HIV-infected individuals, homosexual men, and organ transplant recipients^18^. Interestingly, recent case reports found out the reactivation of several herpesviruses in a small number of COVID-19 patients which supporting this possibility^24–26^. Third, it remains unclear whether pre-existing KSHV may affect SARS-CoV-2 infection, especially because it is a member of the herpesvirus family which can easily establish lifelong infection in a host. We reported here that expression of ACE2, the major receptor of SARS-CoV-2, was increased in AIDS-KS tissues, although it remains unknown how KSHV may regulate ACE2 in infected cells. We previously demonstrated that KSHV de novo infection upregulated the expression of one multifunctional glycoprotein, CD147 (also named as Emmprin or Basigin), which was also strongly expressed in KS tissues^27,28^. Interestingly, CD147 was recently found to be one of co-receptors to facilitate SARS-CoV-2 entry and invasion of host cells^29^. Therefore, it will be interesting to explore the potential association between KSHV and these SARS-CoV-2 receptors or co-receptors in different types of host cells.

## Methods

### Cell culture and reagents

Human iSLK.219 cells are latently infected with a recombinant rKSHV.219 virus and contain a doxycycline (Dox)-inducible RTA, constructed and named by Dr. Don Ganem’s lab^12^. The rKSHV.219 virus expresses both the red fluorescent protein (RFP) under the control of KSHV lytic PAN promoter and the green fluorescent protein (GFP) under the control of the elongation factor 1 promoter (EF-1α)^30^. HEK293T (Human embryonic kidney 293T) cells and KSHV + PEL cell lines, BCP-1 and BCBL-1, were purchased from American Type Culture Collection (ATCC) and cultured as recommended by the manufacturer. The anti-COVID-19 drugs: Azithromycin, Chloroquine diphosphate, Hydroxychloroquine sulfate, Nafamostat mesylate, Remdesivir, and Tocilizumab were purchased from Selleck Chemicals. All the other chemicals if not indicated specifically were purchased from Sigma-Aldrich.

### Plasmids transfection

Human iSLK.219 cells were transfected with recombinant vectors of SARS-CoV-2 spike protein (S), nucleocapsid protein (N) (both purchased from Sino Biological), pCR3.1-RTA (a gift from Dr. Yan Yuan, University of Pennsylvania)^31^, pFLAG-CMV2-p65 (pFLAG-p65, a gift from Dr. Ren Sun, University of California Los Angeles)^32^, or vector controls, using Lipofectamine^TM^ 3000 reagent (Invitrogen). Transfection efficiency was normalized through co-transfection of a lacZ reporter construct and determination of β-galactosidase activity using a commercial β-galactosidase enzyme assay system (Promega).

### qRT-PCR

Total RNA was isolated using the RNeasy Mini kit (Qiagen), and cDNA was synthesized using a SuperScript III First-Strand Synthesis SuperMix Kit (Invitrogen). Primers used for amplification of target genes are listed in Supplementary Table 1. Amplification was carried out using an iCycler IQ Real-Time PCR Detection System, and cycle threshold (Ct) values were tabulated in duplicate for each gene of interest in each experiment. “No template” (water) controls were used to ensure minimal background contamination. Using mean Ct values tabulated for each gene, and paired Ct values for *β-actin* as a loading control, fold changes for experimental groups relative to assigned controls were calculated using automated iQ5 2.0 software (Bio-Rad).

### Cell proliferation assays

Cell proliferation was measured using the WST-1 Assay (Roche). Briefly, after the period of treatment of cells, 10 μL/well of cell proliferation reagent, WST-1 (4-[3-(4-Iodophenyl)-2-(4-nitro-phenyl)-2H-5-tetrazolio]-1,3-benzene disulfonate), was added into 96-well microplate and incubated for 3 h at 37 °C in 5% CO_2_. The absorbance of samples was measured using a microplate reader at 490 nm.

### Western blot

Total cell lysates (20 μg) were resolved by 10% SDS–PAGE, transferred to nitrocellulose membranes, and immunoblotted with antibodies to SARS-CoV-2 S or N (Abcam), KSHV ORF45 (Novus Biologicals), phosphor (p)-p65 (Ser536)/total (t)-p65, p-ERK (Thr202/Tyr204)/t-ERK, p-JNK (Thr183/Tyr185)/t-JNK, p-p38 (Thr180/Tyr182)/t-p38, FLAG and GAPDH or β-Actin as a loading control (Cell Signaling). Immunoreactive bands were identified using an enhanced chemiluminescence reaction (Perkin-Elmer), and visualized by autoradiography. The full, uncropped blot/gel images were listed as Supplementary Figs. 5 and 6, respectively.

### KS tumor tissues from HIV+ patients and immunohistochemistry

KS tissues from HIV-infected patients were provided by the Louisiana State University Health Sciences Center (LSUHSC) HIV Outpatient (HOP) Clinic and Biospecimens Bank. The study was approved by the Institutional Review Board for Human Research (approval no. 8079) at LSUHSC. All subjects provided written informed consent. Formalin-fixed, paraffin-embedded tissues were microtome-sectioned to a thickness of 4 µm, placed on electromagnetically charged slides (Fisher Scientific). Immunohistochemistry was performed as described previously^31^, and the ACE2 antibody was purchased from Abcam. Images were collected using an Olympus BX61 microscope equipped with a high-resolution DP72 camera and CellSense image capture software.

### Statistics and reproducibility

All statistics were performed using Graphpad Prism v. 8.0. Normality and variance were evaluated before performing statistical analysis. Significance for differences between experimental and control groups was determined using the two-tailed Student’s *t*-test. Number of repeated experiments was described in the figure legends. Repeated experiments produced comparable results and findings were considered reproducible.

### Reporting summary

Further information on research design is available in the Nature Research Reporting Summary linked to this article.

## Supplementary information

Supplementary Information

Description of Additional Supplementary Files

Supplementary Data 1

Reporting Summary

## Data Availability

All relevant data are within the manuscript and its supplementary files. Source data for figures are available in Supplementary Data 1. In addition, data that support the findings of this study are available from the corresponding author (Z.Q.) upon reasonable request.
