# Supplementary material for: SARS-CoV-2 proteins and anti-COVID-19 drugs induce lytic reactivation of an oncogenic virus
Source: Commun Biol. 2021 Jun 3;4:682. doi: 10.1038/s42003-021-02220-z (PMC8175744; doi:10.1038/s42003-021-02220-z)
Supplement: Supplementary file 1 — Supplementary Information [file 42003_2021_2220_MOESM1_ESM.pdf]

Supplementary Table 1. Primer sequences for qRT-PCR.

Gene	Sequences (5' → 3')
LANA	*sense TCCCTCTACACTAAACCCAATA*
	antisense TTGCTAATCTCGTTGTCCC
RTA	*sense TAATGTCAGCGTCCACTCC*
	antisense TTCTGGCACGGTCAAAGC
ORF59	*sense CGAGTCTTCGCAAAAGGTTC*
	antisense AAGGGACCAACTGGTGTGAG
ORF17	*sense AGATTTTTCACGGGGGCTCTGG*
	antisense TGGGCTGGACACTGGGTCTATTTC
β-actin	*sense GGAAATCGTGCGTGACATT*
	antisense GACTCGTCATACTCCTGCTTG

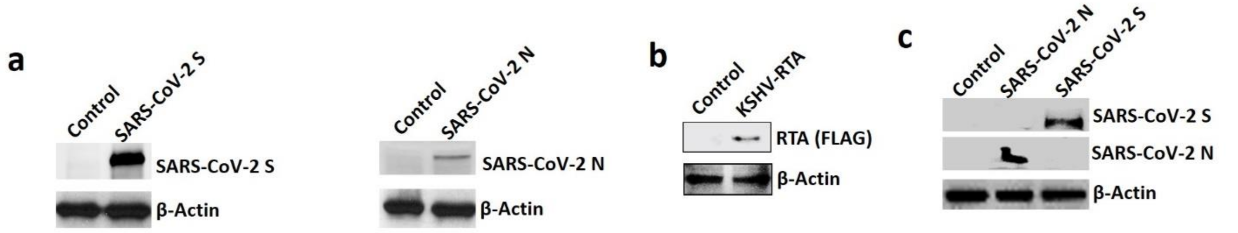

Supplementary Figure 1. Ectopic expression of SARS-CoV-2 proteins in KSHV latently infected cells. (**a-b**) The iSLK.219 cells were transfected with vector control or vectors encoding SARS-CoV-2 spike protein (S), nucleocapsid protein (N), KSHV-RTA for 72 h, then protein expression was detected using Western blot. (**c**) The BCP-1 cells were transfected as above, then protein expression was detected using Western blot. Representative blots from one of two independent experiments were shown.

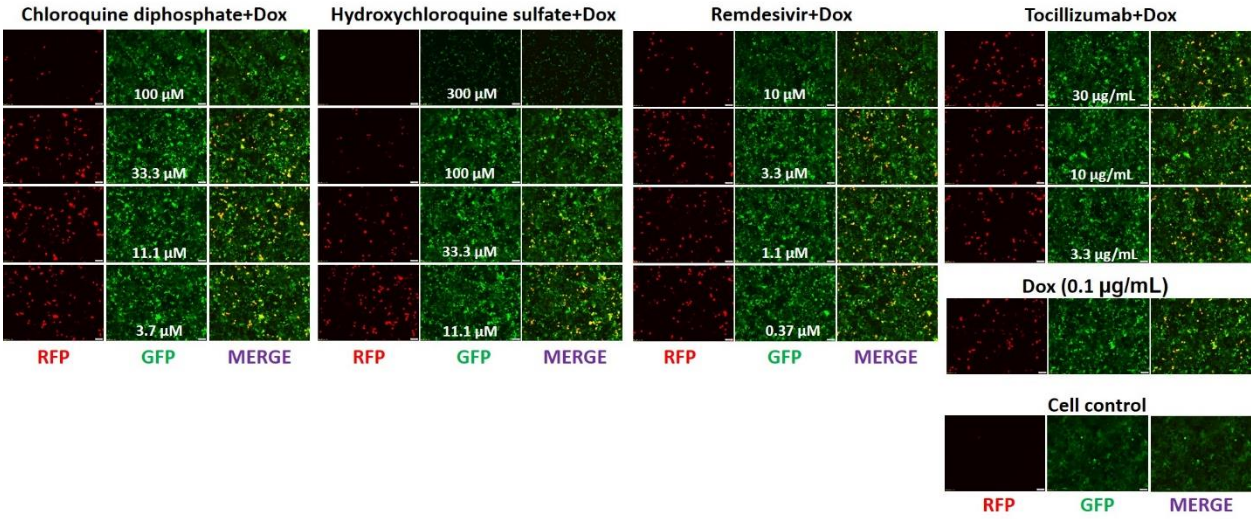

Supplementary Figure 2. The impacts of anti-COVID-19 drugs on KSHV lytic reactivation. The iSLK.219 cells were treated with a dose range of anti-COVID-19 drugs together with doxycycline (Dox, 0.1 μg/mL) induction for 72 h. The expression of RFP and GFP were detected using fluorescence microscopy.

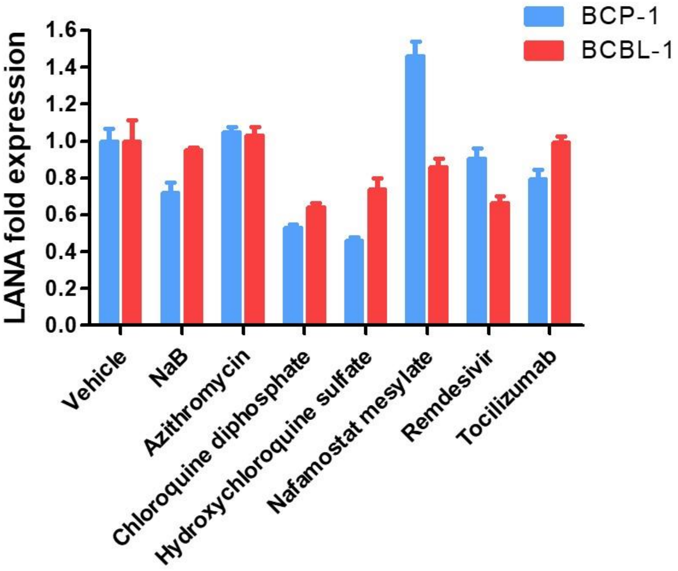

Supplementary Figure 3. The impacts of anti-COVID-19 drugs on viral latent gene expression from KSHV+ tumor cells. BCBL-1 and BCP-1 cells were treated with Azithromycin (10 μM), Chloroquine diphosphate (10 μM), Hydroxychloroquine sulfate (10 μM), Nafamostat mesylate (10 μM), Remdesivir (3 μM), Tocilizumab (20 μg/mL), respectively, for 72 h, then the transcripts of representative latent gene, *Lana*, were quantified by using qRT-PCR. The sodium butyrate (NaB, 0.3 mM) was used as a positive control. Error bars represent S.D. for 3 independent experiments.

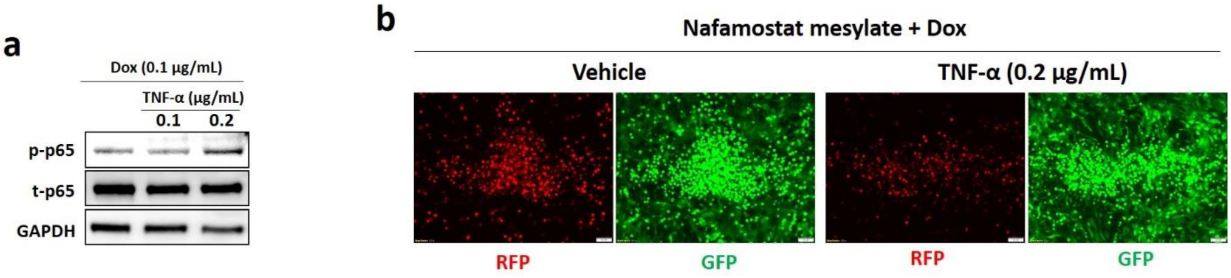

Supplementary Figure 4. Pre-treatment of TNF-α blocks Nafamostat mesylate induced KSHV lytic reactivation. (**a-b**) The iSLK.219 cells were pre-treated with TNF-α for 12 h, then addition of Nafamostat mesylate together with doxycycline (Dox, 0.1 μg/mL) induction for 72 h. The effects of TNF-α on signaling activities were confirmed by immunoblots. Representative blots from one of two independent experiments were shown. The expression of RFP and GFP were detected using fluorescence microscopy.

Figure3C

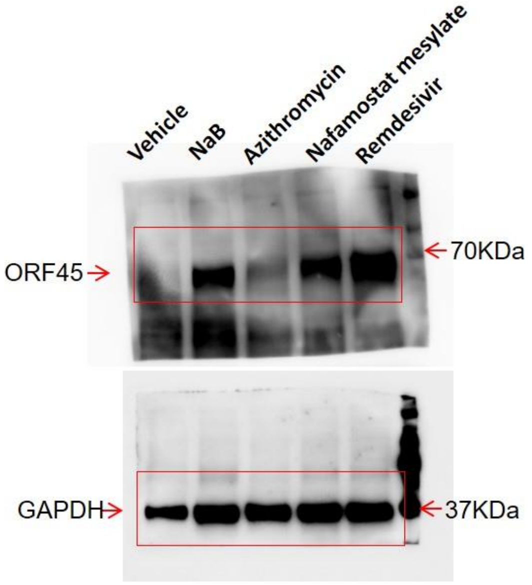

Supplementary Figure 5. Original immunoblots of Figure 3c.

Figure4A

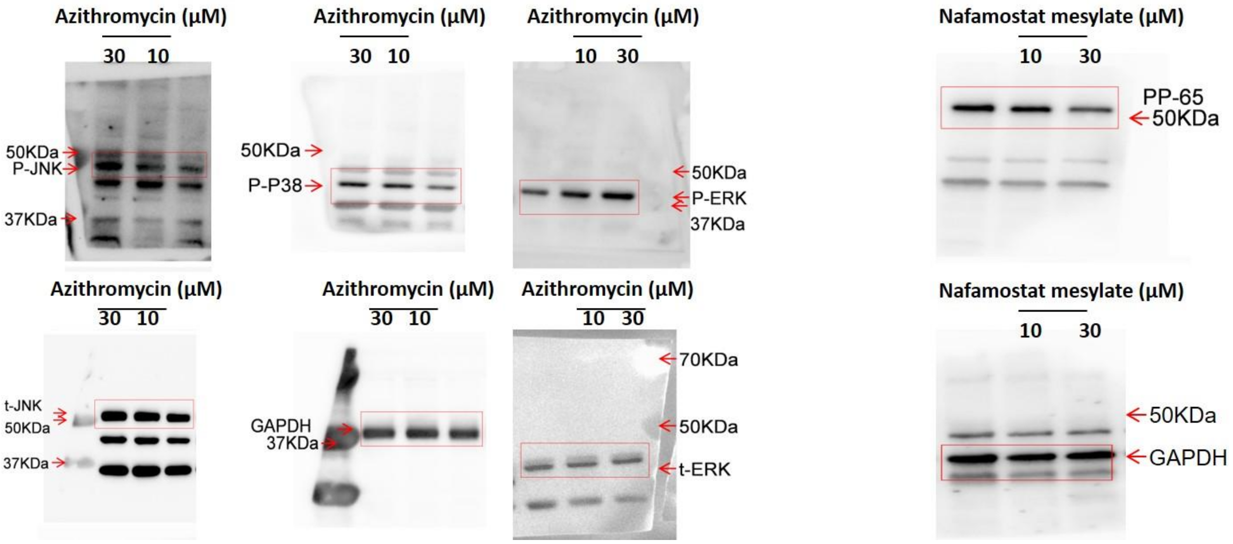

Figure4D

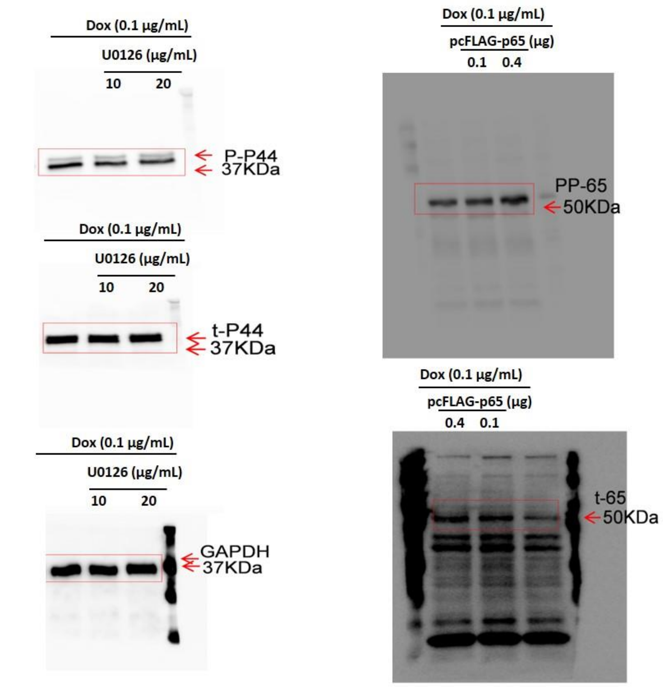

Supplementary Figure 6. Original immunoblots of Figure 4a and 4d.
