# Supplementary material for: SARS-CoV-2 proteins and anti-COVID-19 drugs induce lytic reactivation of an oncogenic virus
Source: Commun Biol. 2021 Jun 3;4:682. doi: 10.1038/s42003-021-02220-z (PMC8175744; doi:10.1038/s42003-021-02220-z)
Supplement: Supplementary file 4 — Reporting Summary [file 42003_2021_2220_MOESM4_ESM.pdf]

nature research

Corresponding author(s): Zhiqiang Qin

Last updated by author(s): Apr 24, 2021

Reporting Summary

Nature Research wishes to improve the reproducibility of the work that we publish. This form provides structure for consistency and transparency in reporting. For further information on Nature Research policies, see our Editorial Policies and the Editorial Policy Checklist.

Statistics

For all statistical analyses, confirm that the following items are present in the figure legend, table legend, main text, or Methods section.

n/a	Confirmed	
☐	☒	The exact sample size (*n*) for each experimental group/condition, given as a discrete number and unit of measurement
☐	☒	A statement on whether measurements were taken from distinct samples or whether the same sample was measured repeatedly
☐	☒	The statistical test(s) used AND whether they are one- or two-sided<br>*Only common tests should be described solely by name; describe more complex techniques in the Methods section.*
☒	☐	A description of all covariates tested
☒	☐	A description of any assumptions or corrections, such as tests of normality and adjustment for multiple comparisons
☐	☒	A full description of the statistical parameters including central tendency (e.g. means) or other basic estimates (e.g. regression coefficient) AND variation (e.g. standard deviation) or associated estimates of uncertainty (e.g. confidence intervals)
☐	☒	For null hypothesis testing, the test statistic (e.g. *F*, *t*, *r*) with confidence intervals, effect sizes, degrees of freedom and *P* value noted<br>*Give P values as exact values whenever suitable.*
☒	☐	For Bayesian analysis, information on the choice of priors and Markov chain Monte Carlo settings
☒	☐	For hierarchical and complex designs, identification of the appropriate level for tests and full reporting of outcomes
☒	☐	Estimates of effect sizes (e.g. Cohen's *d*, Pearson's *r*), indicating how they were calculated

Our web collection on statistics for biologists contains articles on many of the points above.

Software and code

Policy information about availability of computer code

Data collection	No software was used
Data analysis	Excel 2016 was used for data analysis

For manuscripts utilizing custom algorithms or software that are central to the research but not yet described in published literature, software must be made available to editors and reviewers. We strongly encourage code deposition in a community repository (e.g. GitHub). See the Nature Research guidelines for submitting code & software for further information.

Data

Policy information about availability of data

All manuscripts must include a data availability statement. This statement should provide the following information, where applicable:

- Accession codes, unique identifiers, or web links for publicly available datasets
- A list of figures that have associated raw data
- A description of any restrictions on data availability

All relevant data are within the manuscript and its supplemental files.

Field-specific reporting

Please select the one below that is the best fit for your research. If you are not sure, read the appropriate sections before making your selection.

☒ Life sciences ☐ Behavioural & social sciences ☐ Ecological, evolutionary & environmental sciences

For a reference copy of the document with all sections, see nature.com/documents/nr-reporting-summary-flat.pdf

Life sciences study design

All studies must disclose on these points even when the disclosure is negative.

Sample size	No sample size calculation was performed, because it is not relevant to our study.
Data exclusions	No data were excluded from these analyses.
Replication	The replication of different kinds of experiments have been explained in figure legends.
Randomization	This is not relevant to our study.
Blinding	The investigators were blinded to group allocation during data collection and/or data analysis.

Reporting for specific materials, systems and methods

We require information from authors about some types of materials, experimental systems and methods used in many studies. Here, indicate whether each material, system or method listed is relevant to your study. If you are not sure if a list item applies to your research, read the appropriate section before selecting a response.

Materials & experimental systems

n/a	Involved in the study	
☐	☒	Antibodies
☐	☒	Eukaryotic cell lines
☒	☐	Palaeontology and archaeology
☒	☐	Animals and other organisms
☒	☐	Human research participants
☒	☐	Clinical data
☒	☐	Dual use research of concern

Methods

n/a	Involved in the study	
☒	☐	ChIP-seq
☒	☐	Flow cytometry
☒	☐	MRI-based neuroimaging

Antibodies

Antibodies used	The antibodies to SARS-CoV-2 S (#ab272504) or N (#ab273167) (Abcam, Cambridge, MA, USA), phosphor (p)-p65 (#3033)/total (t)-p65 (#4764), p-ERK (#4370)/t-ERK (#4695), p-JNK (#4668)/t-JNK (#9258), p-p38 (#4511)/t-p38 (#9212) and GAPDH (#5174) as a loading control (Cell Signaling, Danvers, MA, USA).
Validation	The validation of these commercial antibodies are obtained from the vendors' website.

Eukaryotic cell lines

Policy information about cell lines

Cell line source(s)	Human iSLK.219 cells were constructed and obtained from Dr. Don Ganem's lab (Ref. 12). HEK293T (Human embryonic kidney 293T) cells and KSHV+ PEL cell lines, BCP-1 and BCBL-1, were purchased from American Type Culture Collection (ATCC).
Authentication	The authentication of HEK293T, BCP-1, BCBL-1 can be found on ATCC website; the details about the creation of iSLK.219 can be found in Ref. 12.
Mycoplasma contamination	All the cell lines were tested negative for mycoplasma contamination.
Commonly misidentified lines (See ICLAC register)	n/a
